# Performance measurement for co-occurring mental health and substance use disorders

**DOI:** 10.1186/1747-597X-4-18

**Published:** 2009-10-14

**Authors:** David J Dausey, Harold A Pincus, James M Herrell

**Affiliations:** 1 Carnegie Mellon University, Pittsburgh PA 15213, USA; 2 RAND Corporation, Pittsburgh PA 15213, USA; 3 College of Physicians and Surgeons, Columbia University, and New York Presbyterian Hospital and Irving Center for Clinical and Translational Research, New York NY 10032, USA; 4 Substance Abuse and Mental Health Services Administration (SAMHSA)/Center for Substance Abuse Treatment (CSAT), Rockville, MD 20850, USA

## Abstract

**Background:**

Co-occurring mental health and substance use disorders (COD) are the norm rather than the exception. It is therefore critical that performance measures are developed to assess the quality of care for individuals with COD irrespective of whether they seek care in mental health systems or substance abuse systems or both.

**Methods:**

We convened an expert panel and asked them to rate a series of structure, process, and outcomes measures for COD using a structured evaluation tool with domains for importance, usefulness, validity, and practicality.

**Results:**

We chose twelve measures that demonstrated promise for future pilot testing and refinement. The criteria that we applied to select these measures included: balance across structure, process, and outcome measures, quantitative ratings from the panelists, narrative comments from the panelists, and evidence the measure had been tested in a similar form elsewhere.

**Conclusion:**

To be successful performance measures need to be developed in such a way that they align with needs of administrators and providers. Policymakers need to work with all stakeholders to establish a concrete agenda for developing, piloting and implementing performance measures that include COD. Future research could begin to consider strategies that increase our ability to use administrative coding in mental health and substance use disorder systems to efficiently capture quality relevant clinical data.

## Background

There has been a dramatic growth in recent years in the development of performance measures to monitor and evaluate the quality of medical care [1]. Examples include performance measures developed for the Institute of Medicine's (IOMs) national report card [2], the National Committee for Quality Assurance's (NCQA) Healthcare Effectiveness Data and Information Set (HEDIS) [3], the National Quality Forum's (NQF) "Standardizing Ambulatory Care Performance Measures" project [4], and the American Medical Association's outpatient chronic care clinical performance measures [5].

These measures have been used for everything from internal auditing and quality improvement efforts to benchmarking performance against national averages. Most recently, performance measures are being used to financially reward performance with pay-for-performance initiatives and to distinguish between high and low performing health care systems [6-8]. The substance abuse and mental health fields have long used performance measures such as length of stay, readmission rates, and abstinence during drug treatment. Despite this, the growth of performance measure development in these fields has not kept pace with some other sectors of medicine [9,10]. As with other fields, this interest has been motivated by calls to increase transparency and accountability and thereby improve the overall quality of services delivered [11-13].

The limited numbers of performance measures available tend to focus on either mental health disorders (MHD) [14,15] or substance use disorders (SUD) [16,17], despite the fact that these disorders have a high probability of co-occurrence. This reflects the segmentation of mental health and substance abuse services into distinct clinical and organizational "silos" [18].

The idea of developing performance measures for co-occurring conditions that cut across these silos is still in its infancy [19]. A notable effort by researchers to develop such measures is the Substance Abuse and Mental Health Services Administration's (SAMHSA) National Outcome Measures (NOMs) project [20]. NOMs comprise ten outcome domains conceptualized to apply to a variety of populations, including persons with COD. Operationalzing NOMs into concrete, measurable performance indicators, however, is still necessary.

SAMSHA contracted with the RAND Corporation in 2006-2007 to begin to conceptualize and develop performance measures for COD. This pilot effort was focused on COD performance measures specifically for SUD settings. However, we discuss how these measures can be easily adapted to be used in MHD settings. The project was designed to rely on the advice of an expert panel to aid in the assessment of a set of candidate COD performance measures. Our goal was to develop a small number of measures that could serve as examples of the types of measures for COD that could be pilot tested and refined for future use. Here we outline the process we used to develop these measures and describe the final set of measures that were developed.

## Methods

We convened an expert panel that included a range of stakeholders. Our goal was to include the diverse perspectives of different groups involved in performance measurement in substance abuse and mental health. To this end, we first identified three broad perspectives to include on the panel: (1) state and local perspectives (e.g., individuals with leadership roles related to performance measurement at state and local substance abuse and mental health agencies and national substance abuse and mental health professional associations); (2) health plan perspectives (e.g., individuals who have a role in performance measurement at behavioral health plans); and (3) academic perspectives (e.g., experts from leading academic and research centers who have published on this topic).

We identified a convenience sample of more than thirty experts with backgrounds in at least one of these perspectives and asked them to participate in our panel. Of those contacted, 18 agreed to participate. There was an even distribution of panelists from these perspectives. Based on their career experience, some panelists were able to consider the measures from multiple perspectives. Panelists were asked to respond to a series of quarterly questionnaires over the course of one year. The first two questionnaires asked participants to evaluate the general framework we developed. The remaining questionnaires asked participants to evaluate a three sets of performance measures (structure, process, and outcome) using a systematic data collection form described below. A majority of panelists responded to each questionnaire.

At the start of the project, we asked the panelists to evaluate the basic framework we established to conceptualize, develop, and evaluate our performance measures. The primary conceptual backdrop for the development of the measures was Donabedian's classic triad of structures, processes, and outcomes [21]. We proposed using this framework to categorize our measures.

On the advice of the panelists, we modified this approach by creating subcategories for each part of the triad: (1) structure (e.g., clinician characteristics, clinical information systems, service linkages and financial); (2) process (e.g., detection/identification, assessment, treatment, and service integration/coordination of care) and (3) outcomes (e.g., reduced morbidity, crime and criminal justice, stability in housing, social connectedness, and perception of care).

The subcategories for the structure and process measures were developed from the integration of information derived from the literature, suggestions from the panelists, and suggestions we received during a presentation on this topic at a professional meeting [22]. To ensure consistency with ongoing SAMHSA work in this area, the subcategories for the outcomes measures were derived from the NOMs project.

In developing the performance measures we considered existing work in this area including: (1) performance indicators being developed for the Performance Partnership Grants-PPG [23]; (2) local evaluations of individual Co-Occurring State Incentive Grant (COSIG) projects [24]; (3) quality indicators for substance abuse services developed by the Washington Circle (WC) Group [25]; (4) agency for Healthcare Research and Quality (AHRQ) quality measure clearinghouse [26]; (5) mental health quality indicators developed for the Healthcare Effectiveness Data and Information Set (HEDIS) [3]; and (6) quality indicators identified and/or developed by the Center for Quality Assessment and Improvement in Mental Health (CQAIMH) [27].

Our goal was to develop measures that built on existing work. We started with the assumption that existing performance measures in the substance abuse and mental health fields could be modified to apply to COD. Despite this, we did not want to be constrained to what has already been done. Therefore in addition to adapting existing performance measures to COD, we also developed novel measures that we considered to be of potentially high value.

The candidate measures are meant to serve as exemplars of the types of measures that can be developed. Therefore, some candidate measures specify a particular setting (e.g., inpatient) when they could potentially apply to more than one setting depending on the circumstance. In addition, despite the fact that the measures developed were designed to apply to substance abuse settings, they could easily be adapted to apply to mental health settings.

We asked the panelists to provide feedback on the framework we established for evaluating the measures themselves. We adapted the initial performance measure evaluation tool from one developed by Hermann and colleagues which was provided to the first author electronically (see [27] for more information on Hermann's work). The tool was a simple rating form divided along 6 domains ranging from clinical importance to strategic importance. All of the ratings consisted of 5 point Likert scales. The descriptions of the domains paralleled NCQAs list of desirable attributes for HEDIS measures [3].

We adapted this tool by limiting it to five domains and adapting the descriptions of these domains to apply to COD. We asked the panelists to evaluate our choice of domains and our descriptions of them, and they provided us with feedback on how to improve them. Below we list the final set of domains that we developed based on the feedback from our panelists:

• ***Importance ***- Does the measure represent a significant part of the overall quality picture? Does the measure represent an aspect of COD care that is meaningful (e.g., does what is being measured represent a significant quality deficit)?

• ***Usefulness ***- Will the use of the measure be helpful in leading to strategies that will result in improvements in patient care? Is the measure likely to have a significant impact on patient care and have the ability to lead to real change in patient outcomes (e.g., is the deficit potentially correctable by an identifiable group)?

• ***Validity or Scientific Soundness ***- Is the measure evidence based, is it sensitive to case mix or can it be adjusted to case mix? Can it be gamed or manipulated? Is the measure something that can quantitatively demonstrate changes over time (e.g., if the process is corrected, would the measure be sensitive to detecting this change)?

• ***Practicality or Feasibility ***- How practical is it to collect information for the measure (e.g., will data collection require significant costs or be overly burdensome with existing data systems)?

• ***Overall ***- What is your overall impression of this measure? Should this measure be included in the core measure set?

When rating the measures, panelists were given detailed descriptions of each measure and asked to rate the measures on each of the domains listed above using a Likert scale where 1 represented strongly agree, 3 represented uncertain, and 5 represented strongly disagree. Space was also provided for panelists to provide narrative comments on each of the measures or to propose additional measures. We also encouraged the panelists to use the space for narrative comments to consider the most appropriate audience for each of the proposed measures. In our initial questionnaire to the panelists we provided several potential examples including: purchasers (public and private), health Plans, agencies/provider organizations, individual providers, consumers, and policymakers.

We developed a total of 36 performance measures broken into structures, processes, and outcomes with at least one measure for each subcategory identified above. We chose a total of twelve measures (four from each category--structure, process, and outcome) that appeared to have the most promise to pilot test. The criteria that we applied to choose the set of twelve measures included: balance across structure, process, and outcome measures, quantitative ratings from the panelists, narrative comments from the panelists, and evidence the measure had been tested in a similar form elsewhere.

## Results

### Structure measures

Structure measures tell us whether or not a health care agency or organization has the capacity for process or outcomes measurement (e.g., infrastructure in place that enables quality measurement to occur) and in the case of COD whether or not any integrated or linked services are offered. To measure the quality of COD care, an agency must first have the capacity to deliver integrated or linked services. Structural measures are designed to enable us to access these capacities. Table 1 presents our final refined set of structure measures [see Additional file 1].

Measure 1 assesses the proportion of SUD providers in a SUD specialty care setting who are trained to provide specified mental health care, and who have a certificate, license or some other documentation to demonstrate proficiency. Data for this measure could be collected with a facility survey. A similar measure could be created for MHD providers by assessing the proportion of MHD providers in MHD specialty care settings who have documented proficiency in SUD care.

Measure 2 assesses the proportion of programs in a defined service area (e.g., county, city or state) that report having integrated services (e.g., SUD and MHD services in the same treatment program) or co-located services (e.g., SUD and MHD services in the same location). Program records used to develop this measure could be augmented by the use of standardized instrument measures of fidelity to the integrated services program--see [28] for an example.

Measure 3 assesses the proportion of SUD providers in a defined service area (e.g., county, city or state) reporting the ability to bill for MHD services provided to patients. A similar measure could also be developed for MHD providers providing SUD services where there are also challenges related to billing for COD services.

Measure 4 assesses the proportion of SUD specialty care settings in a defined service area (e.g., county, city or state) that have formal documented referral policies for MHD services. Data for this measure could be collected by a facility survey that could be auditable by facility records.

Measures 1-4 have the potential to be useful for state and local SUD and MHD administrators evaluating the level of integration or the quality of the linkages between SUD and MHD agencies in their jurisdiction.

### Process measures

After we have determined that an agency is capable of measuring performance and that it has some infrastructure in place to deliver integrated or linked services for COD, a logical next step is to consider process measures that examine how care is being delivered. Process measures are a critical link in the chain between structure measures and outcome measures because they enable us to determine whether or not care is being delivered using evidence based standards and guidelines. Without knowing this, it isn't possible to know whether poor outcomes are the result of inadequate program implementation, poor program fidelity, or other factors. Table 2 presents our final refined set of process measures [see Additional file 2].

Measure 5 assesses the proportion of individuals formally screened for a MHD upon admission to a SUD specialty care setting. This measure lends itself to be initially piloted in an inpatient setting where standardized charts are more likely. Collecting data for the measure may require the addition of a field to initial patient intake forms. Importantly, the data collected for this measure could be used for denominator data for other measures.

Measure 6 assesses the proportion of individuals that screened positive for COD in a SUD specialty care setting that received a MHD service (or at least one integrated service) within 30 days of screening. This measure is adapted from measures developed by the Washington Circle Group for substance abuse settings [16] which were pilot tested in six states by state and local substance abuse and/or mental health agencies [17]. The pilot test revealed that state agencies could calculate these types of measures with routinely collected data. A measure such as this might be useful for state-level SUD agencies and might encourage them to develop the structural capacity to measure COD screening.

Measure 7 assesses the proportion of COD with an inpatient or day/night episode (SUD or MHD related) visit that have at least one SUD and one MHD outpatient clinic visit (or one integrated treatment visit) within thirty days of discharge. This measure was adapted from a HEDIS measure for mental illness [29]. The original HEDIS measure was found to be moderately correlated with some but not all similar measures of outpatient performance [30]. Data collection for this measure may require chart reviews in some systems. The denominator data could come from the total number of positive screens found with Measure 5 above.

Measure 8 assesses the proportion of individuals with COD that were assessed for housing stability. A measure such as this could be transitioned into an administrative dataset. The method of identification, however, needs to specify whether it is based on positive screens or based on receipt of both types of services or integrated services. Ultimately it may be possible to develop specific administrative codes to integrate this type of information into administrative datasets.

Measures 5-8 have the potential to be useful to specialty plan administrators to assess the level of integrated or linked services being offered in their plan.

### Outcome measures

Outcomes are the final piece of the performance measurement strategy. Determining what outcomes to measure, however, is not always easy. Different outcomes are important to different stakeholders. For example, patients may be most interested in outcomes that deal with improvements to their quality of life or that improve their functionality while policymakers may be interested in outcomes that reduce crime or inpatient costs. Table 3 presents our final refined set of outcomes measures [see Additional file 3]. All of these measures were based on generic outcomes measures included in NOMs.

Measure 9 assesses the proportion of individuals with any MHD discharged from an inpatient or residential SUD specialty care setting with abstinence from drugs and/or alcohol one year after discharge. The data for this measure could come from patient report and/or laboratory tests. This measure focuses on inpatient care; however, it is possible for a similar measure to be developed for outpatient care.

Measure 10 assesses the proportion of individuals with any MHD diagnosis discharged from an inpatient or residential SUD specialty setting that move from being unemployed to being employed either part-time or full-time one year after discharge. The data from this measure could come from patient report and/or employment records. As with Measure 9, a similar measure could be developed for outpatient settings.

Measure 11 assesses the proportion of individuals with any MHD diagnosis discharged from an inpatient or residential SUD specialty care setting who report having an episode of incarceration within 6 months of discharge. Data for this measure could come from patient report and/or criminal justice system data. As with Measures 9-10, a similar measure for outpatient settings could also be developed.

Measure 12 assesses the proportion of individuals receiving care in a SUD specialty care setting with any MHD diagnosis who report improved satisfaction with their care as measured by a standardized instrument after 6 months of treatment. Data for this measure could come from satisfaction measures included on patient surveys. The respondent pool for these surveys could be determined by claims data or a checkbox for self-report of a MHD.

Measures 9-12 have the potential to be useful to SUD and MHD providers to assess how well they are providing integrated or linked SUD and MHD services.

## Discussion

Developing reliable and valid performance measures for COD that are of high value while remaining feasible to implement is a significant challenge. This project sought to take a step forward in the development of such measures by developing a framework to conceptualize these measures and by considering a small set of measures for substance abuse settings evaluated through expert consensus. The model introduced by this project for initially developing performance measures for COD is a logical first step and could be expanded and replicated elsewhere.

Because of the qualitative nature of our analysis, we do not present the raw data from our analysis of the expert panelist feedback. This feedback was provided in the form of ratings using Likert scales in combination with written qualitative feedback for each measure. It is important to note, however, that overall our panelists rated outcomes measures as more important than structure and process measures. This likely reflects the general belief that outcomes are the ultimate aspect of quality that we are trying to assess in any performance measurement system. Despite this, in choosing our final set of measures, we purposely included an equal number of structure, process, and outcomes. We agree with the importance of outcomes measures; however, we argue that these measures need to be part of an integrated package of measures with the intent for full implementation over time. To interpret outcomes and to use outcomes to improve service, measures of structure and process are essential.

A tension in performance measurement is the extent to which measures can be developed using existing administrative data systems versus those that require additional data collection. Using administrative data is less costly and involves less effort; however, these measures tend to be "black box" measures that don't really allow us to directly assess what we want to measure. Developing new data systems or additional data collection applets in old data systems can be costly and if done without proper foresight can yield limited benefits. It can be argued, however, that without considering new or modified data systems that we will never be able to measure quality adequately.

For many psychosocial problems (e.g., homelessness, unemployment, family interventions, legal problems), without having a simple and efficient way of identifying them (i.e., identifying who needs help) it is difficult to develop performance measures that do not require extensive chart reviews. This is less of a problem with developing performance measures so much as it is a problem with making the data collection for these measures feasible. A coding system to capture this information that could be integrated into the clinical record is one way to solve this problem [31].

This project has several limitations. In choosing the measures, we tried to achieve a balance between attributes with a particular emphasis on practicality. There are many other measures that could be developed. The measures presented here are intended to serve as exemplars of the types of measures that could be developed. They also serve as measures that are potentially promising to pilot test.

The setting in which each measure is most applicable varies (e.g., health plans, SUD organizations, providers). Our emphasis was to focus on settings that were most likely to have data. That data for some of these measures could become unstable for smaller organizations. Nonetheless the measure sets a clear goal and can aid even these small organizations in moving towards that goal.

Several of the measures that we developed focused on inpatient care settings. This emphasis is not meant to suggest that outpatient care is not important. More outpatient measures will need to be developed over time. Inpatient measures; however, provide us with an environment where we have the most control and provide us with a date to start counting. In addition, appropriate follow-up after inpatient care is a high priority.

These measures and others that may be developed in the future will need to be continually refined as we gain an understanding of their psychometric and statistical properties. Refinements will need to include weighting procedures and other statistical adjustments. For example, Measure 3 assesses the proportion of SUD providers within a geographical area that are able to bill for MHD services. Compared to community based SUD providers, hospital based SUD providers may be more likely to be able to bill for MHD services while at the same less likely to see as many clients. Thus areas with higher proportions of hospital based providers compared to community based providers could score higher on this measure while treating fewer COD clients than areas with the reverse. Strategies to appropriately adjust for caseload will be needed to solve these types of problems.

This project raises several avenues for future research in this area. A logical next step is to pilot test these measures (or other measures that are developed) in real settings. These pilot tests could take on one of two forms: pilot tests of measures that lend themselves to existing administrative datasets versus pilot tests of measures that require additional data collection. Existing administrative datasets of one or more health care organizations can used to test the feasibility of the current specifications of the measures. Modifications can be made to these specifications based on what is learned. It may turn out that some of the measures that initially seemed feasible with administrative data are less amenable to this format than originally thought. Measures that involve data not contained in administrative or claims datasets will require additional data collection. These measures can be pilot tested in practice care settings through surveys of patients, providers, and family members.

## Conclusion

Having clear processes for developing, vetting and piloting performance measures affords us an opportunity to think about what we want to measure and contrast that with what we are able to measure with existing data systems and infrastructure. Policymakers have a vast array of considerations to take into account when choosing performance measures. They must consider the implementation of new performance measures against the backdrop of scarce resources and competing priorities. New performance measures are critical but may not be possible without additional resources. It is important that as this process moves forward that all stakeholders in the field have an opportunity to review and comment on new performance measures being considered. To be successful performance measures need to be developed in such a way that they align with needs of administrators and providers. Policymakers need to work with all stakeholders to establish a concrete agenda for developing, piloting and implementing performance measures that include COD.

## Competing interests

The authors declare that they have no competing interests.

## Authors' contributions

DJD and HAP developed the measures and conducted the background research. DJD took primary responsibility for writing all sections of the paper. JMH supervised the work and helped to write the background and conclusion sections of the paper. All authors read and approved the final manuscript.

## Supplementary Material

Additional file 1 **Table 1: Candidate Structure Measures**. This table presents all of the candidate structure measures. Click here for file

Additional file 2 **Table 2: Candidate Process Measures**. This table presents all of the candidate process measures. Click here for file

Additional file 3 **Table 3: Candidate Outcome Measures**. This table presents all of the candidate outcome measures. Click here for file
