# Supplementary material for: Performance measurement for co-occurring mental health and substance use disorders
Source: Subst Abuse Treat Prev Policy. 2009 Oct 14;4:18. doi: 10.1186/1747-597X-4-18 (PMC2770527; doi:10.1186/1747-597X-4-18)
Supplement: Additional file 1 — Table 1: Candidate Structure Measures. This table presents all of the candidate structure measures. [file 1747-597X-4-18-S1.DOC]

**Table 1: Candidate Structure Measures***

| **Measure Description** | **Denominator** | **Numerator** | **Data Source** |
| --- | --- | --- | --- |
| **1.** Assesses the proportion of SUD providers in a SUD specialty care setting who are trained to provide specified mental health care, and who have a certificate, license or some other documentation to demonstrate proficiency. | Total number of SUD providers in a SUD specialty care setting | Total number of providers in the denominator with a certificate, license or other acceptable documentation to prove their competency to provide specified mental health care | Facility data |
| **2.** Assesses the proportion of programs in a defined service area (e.g., county, city or state) that report having integrated services (e.g., SUD and MHD services in the same treatment program) or co-located services (e.g., SUD and MHD services in the same location) | Total number of programs in a defined service area | Total number of programs in the denominator that report having integrated or co-located SUD and MHD services | Program records |
| **3.** Assesses the proportion of SUD providers in a defined service area (e.g., county, city or state) reporting the ability to bill for MHD services provided to patients. | Total number of SUD providers in a defined service area | Total number of SUD providers in the denominator that report the ability to bill for MHD services provided to patients | SUD provider survey |
| **4.** Assesses the proportion of SUD specialty care settings in a defined service area (e.g., county, city or state) that have formal documented referral policies for MHD services. | Total number of SUD specialty care settings in a defined service area | Total number of SUD specialty care settings in the denominator with formal documented referral policies for MHD services | Facility survey |

*Measures 1, 3 and 4 can be modified to be used in mental health settings by exchanging SUD for MHD (vice versa). Measure 2 can be applied to both SUD and MHD settings.
