# Supplementary material for: Performance measurement for co-occurring mental health and substance use disorders
Source: Subst Abuse Treat Prev Policy. 2009 Oct 14;4:18. doi: 10.1186/1747-597X-4-18 (PMC2770527; doi:10.1186/1747-597X-4-18)
Supplement: Additional file 2 — Table 2: Candidate Process Measures. This table presents all of the candidate process measures. [file 1747-597X-4-18-S2.DOC]

**Table 2. Candidate Process Measures***

| **Measure Description** | **Denominator** | **Numerator** | **Data Source** |
| --- | --- | --- | --- |
| **5.** Assesses the proportion of individuals formally screened for a MHD upon admission to a SUD specialty care setting | Total number of individuals admitted to a SUD specialty care setting | Total number of individuals in the denominator screened for a MHD upon admission | Administrative/claims datasets and medical records |
| **6.** Assesses the proportion of individuals that screened positive for COD in a SUD specialty care setting that received a MHD service (or at least one integrated service) within 30 days of screening | Total number of individuals served by a substance abuse agency that screened positive for COD | Total number of individuals in the denominator that received a MHD service (or at least one integrated service) within 30 days of the screening | Administrative/claims datasets or chart review |
| **7.** Assesses the proportion of COD with an inpatient or day/night episode (SUD or MHD related) visit that have at least one SUD and one MHD outpatient clinic visit (or one integrated treatment visit) within 30 days of discharge | Total number of COD patients with an inpatient or day/night episode (SUD or MHD related) | Total number of COD patients in the denominator whose medical records indicate that at least one SUD and one MHD outpatient clinic visit (or one integrated treatment visit) within 30 days of discharge | Administrative/claims datasets |
| **8.** Assesses the proportion of individuals with COD that were assessed for housing stability | Total number of individuals identified as having a COD | Total number of individuals in the denominator that were assessed for housing stability | Chart review |

*Measures 5 and 6 can be modified to be used in mental health settings by exchanging SUD for MHD (vice versa). Measures 7 and 8 can be applied to both SUD and MHD settings.
