# Supplementary material for: Performance measurement for co-occurring mental health and substance use disorders
Source: Subst Abuse Treat Prev Policy. 2009 Oct 14;4:18. doi: 10.1186/1747-597X-4-18 (PMC2770527; doi:10.1186/1747-597X-4-18)
Supplement: Additional file 3 — Table 3: Candidate Outcome Measures. This table presents all of the candidate outcome measures. [file 1747-597X-4-18-S3.DOC]

**Table 3. Candidate Outcomes Measures***

| **Measure Description** | **Denominator** | **Numerator** | **Data Source** |
| --- | --- | --- | --- |
| **9.** Assesses the proportion of individuals with any MHD discharged from an inpatient or residential SUD specialty care setting with abstinence from drugs and/or alcohol one year after discharge | Total number of individuals discharged from an inpatient or residential SUD specialty care setting with any MHD diagnosis | Total number of individuals in the denominator that report abstinence from drugs and/or alcohol one year after discharge | Patient report and/or laboratory test |
| **10.** Assesses the proportion of individuals with any MHD diagnosis discharged from an inpatient or residential SUD specialty setting that move from being unemployed to being employed either part-time or full-time one year after discharge | Total number of individuals discharged from an inpatient or residential SUD specialty care setting with any MHD diagnosis | Total number of individuals in the denominator that move from being unemployed to being employed either part-time or full-time one year after discharge | Patient survey and/or employment records |
| **11.** Assesses the proportion of individuals with any MHD diagnosis discharged from an inpatient or residential SUD specialty care setting who report having an episode of incarceration within 6 months of discharge | Total number of individuals discharged from an inpatient or residential SUD specialty care setting with any MHD diagnosis | Total number of individuals in the denominator reporting an episode of incarceration within 6 months of discharge | Patient survey and/or criminal justice system data |
| **12.** Assesses the proportion of individuals receiving care in a SUD specialty care setting with any MHD diagnosis who report improved satisfaction with their care as measured by a standardized instrument after 6 months of treatment | Total number of individuals receiving care in a SUD specialty care setting with any MHD diagnosis | Total number of individuals in the denominator who report improved satisfaction with their care after 6 months of treatment | Patient survey |

*All of these measures can be modified to be used in mental health settings by exchanging SUD for MHD (vice versa).
